# Long-term Functional, Sexual, and Cosmetic Outcomes in Adult Patients Who Underwent Hypospadias Repair During Childhood at a Highly Specialized Italian Pediatric Hospital

**DOI:** 10.5152/tud.2024.24005

**Published:** 2024-03-01

**Authors:** Alessia Celeste Bocchino, Andrea Cocci, Marta Pezzoli, Antonio Elia, Luca Landi, Alberto Mantovani, Chiara Cini, Lorenzo Masieri

**Affiliations:** 1 Department of Urology, University of Cagliari, Santissima Trinità Hospital, Cagliari, Italy; 2 Department of Urology, University of Florence, Careggi Hospital, Florence, Italy; 3 Department of Pediatric Urology, University of Florence, Meyer Children Hospital, Florence, Italy

**Keywords:** Hypospadias, functional outcomes, sexual outcomes, cosmetic outcomes, surgical repair of hypospadias

## Abstract

**Objective:**

This study aims to evaluate long-term functional, sexual, and cosmetic outcomes in adult patients who underwent hypospadias repair during childhood at a national highly specialized pediatric hospital.

**Methods:**

Medical records of pediatric patients who had undergone surgical repair of hypospadias between 1993 and 2004 at Meyer Children Hospital of Florence were reviewed. Adult patients were contacted by telephone between July and August 2021 and invited to participate. Long-term surgical outcomes were assessed focusing on complications and reinterventions, and 3 validated questionnaires on urinary function, erectile function, and penile cosmetic appearance were administered.

**Results:**

From January 1993 to December 2004, a total of 799 patients with hypospadias underwent repair surgery. Two hundred thirty-nine patients gave consent to be included in the study. Follow-ups occurred between 17 and 28 years after the first surgery. Most patients had anterior localization of hypospadias (210/239) and associated penile curvature (132/239). The most frequent surgery for hypospadias repair was meatal advancement and glanduloplasty incorporated (MAGPI) (88/239), and the most used surgical treatment for penile curvature was the Nesbit technique (49/132). The complication rate was 27% (65/239) in an average time of 4.7 years, and 48 surgical procedures have been performed to treat them. At follow-up, the mean IPSS was 0.96 ± 1.97, the mean IIEF-5 score was 24.10 ± 1.02, and the mean HOSE score was 15.47 ± 0.45. Patients who underwent reintervention reported a lower IPSS than those who underwent only 1 surgery (0.29 vs. 1.16).

**Conclusion:**

Hypospadias repair during childhood leads to rather normal urinary and sexual function and penile cosmetic appearance in adolescence and adulthood.

**Main Points**

- **Positive Long-Term Outcomes:** Hypospadias repair in childhood yields excellent surgical results, with most patients experiencing favorable urinary, sexual, and cosmetic outcomes into adulthood despite a 27% complication rate.
- **Prevalence of Surgical Techniques:** MAGPI and Nesbit’s technique are common procedures for hypospadias and penile curvature correction, respectively, with potential lower complication rates associated with techniques like TIP.
- **Assessment of Function and Appearance:** Validated questionnaires reveal satisfactory urinary function, sexual function, and penile appearance for most patients post-repair, emphasizing the importance of long-term follow-up and multidisciplinary care teams.

## Introduction

The broad spectrum of congenital genitourinary anomalies has increased its incidence over the last decades, probably due to improved survival rates, the rise of assisted reproductive technologies, and older motherly age.^1^ Therefore, pediatric urology is facing a great challenge in transitional care because, despite proper surgical treatment in childhood, many genitourinary concerns may arise in adolescence or adulthood.

As a consequence, there has been a new emphasis on long-term urinary, sexual, and cosmetic outcomes, highlighting the importance of effective transitioning of patients from pediatric to adult care.^1^

In this background, hypospadias is one of the most common congenital genitourinary anomalies, with an approximate incidence rate of 1/250 newborns, and an unknown etiology. A associated risk factors likely to be genetic, environmental, and/or placental.^2^

Hypospadias is usually characterized by 3 anatomical features: an ectopic localization of the urethral meatus along the ventral penile shaft from the glans to perineum, a ventral penile curvature, and unevenly distributed foreskin.^2^

The location of the displaced urethral orifice serve as the basis for most classifications, which grade between distal-anterior (the most common type, located on the glans or distal shaft of the penis), intermediate–middle (penile), and proximal-posterior hypospadias (penoscrotal, scrotal, and perineal).^3^

Nevertheless, the complexity and severity of this illness may sometimes go beyond the meatus’s physical placement.^3^ Therapeutic decision-making should be influenced also by penile length, shape, glans size, penile curvature, urethral plate quality, and any other genitourinary abnormalities associated.^3^ These may include cryptorchidism (up to 10%), an open processus vaginalis or inguinal hernia (9%-15%), and ambiguous genitalia.^3^

According to current European Pediatric Guidelines, the suggested age range for primary hypospadias correction surgery is 6- 18 (24) months.^3^

Considering the potential complexity of the disorder, a universal surgical standard approach is not feasible, and the available literature describes almost 300 correction techniques.^2^

Overall, to achieve acceptable functional and cosmetic outcomes, the penile curvature must be corrected with the reconstruction of the neo-urethra distally open on the glans and with proper skin coverage of the penile shaft, including single- and two-stage procedures as well as the use of grafts.^3^

Although the short-term complication rate has been thoroughly investigated, studies on long-term outcomes are still rare.^2^ This is probably due to the lack of adequate transition care from adolescence to adulthood and multidisciplinary lifelong care teams, as well as the absence of standardized assessment protocols with validated questionnaires.

Urethral strictures, voiding dysfunctions, recurrent penile curvature, and ejaculation disorders are the main functional complications that these patients may experience.^3^

Nevertheless, not only urinary and sexual dysfunctions can affect men who have undergone hypospadias repair, but also the perception of cosmetic penile appearance can have a detrimental impact on their quality of life, resulting in potential psychosexual issues.^4^

Unless major complications, hypospadias surgery has no anatomical implications that should lead to erectile dysfunction and fertility problems; nevertheless, these conditions have been reported in the literature.^2^

Moreover, although surgeons might be satisfied with their genito-urinary reconstructive surgery, the patient could maintain a desire for a normal penis, resulting in psychological challenges that affect multiple life domains of adolescent male.^4^

Consequently, questionnaires on the evaluation of lower urinary tract symptoms (LUTS), sexual function, and penile cosmetic appearance are becoming increasingly significant for long-term follow-up.^5^

This study aims to evaluate long-term functional, sexual, and cosmetic outcomes in adult patients who underwent hypospadias repair during childhood between 1993 and 2004 at Meyer Children Hospital of Florence.

## Material and Methods

Health Management of Meyer Children Hospital of Florence approved the study protocol (Approval No: 12; Date: 2021) and did not require approval from the Ethics Committee for the conduct of the research. Verbal informed consent was requested from all study participants.

In our observational cross-sectional study, medical records of pediatric patients who had undergone surgical repair of hypospadias between January 1993 and December 2004 at Meyer Children Hospital of Florence were reviewed. The surgeries were performed by different pediatric surgeons but were part of a team with expertise in urology.

Medical data collected from non-digital records included the age of the patient at the time of surgery, type of hypospadias, associated penile curvatures, surgical technique, complications, and re-intervention. The telephone numbers were also gathered in digital systems; if only family contact details were available, they were contacted to obtain the patient’s ones.

At the time of the follow-up, all patients had reached an age of ≥18 years. Between July and August 2021, they were contacted by telephone call and invited to participate anonymously. Each patient was assigned an ID number, and long-term surgical outcomes were assessed via phone interviews focusing on further complications and reinterventions, as well as three validated questionnaires. Urinary function, erectile function, and penile cosmetic appearance were evaluated using the International Prostatic Symptoms Score (IPSS), International Index of Erectile Function-5 (IIEF-5), and Hypospadias Objective Score Evaluation (HOSE) questionnaires, respectively.

Data were checked for normal distribution using the Kolmogorov–Smirnov statistics. Descriptive analysis was reported according to continuous distribution using mean (SD) and percentages to express data. Statistical Package for the Social Sciences (SPSS) version 20.0 (IBM SPSS Corp.; Armonk, NY, USA) was used for statistical anaylsis.

## Results

From January 1993 to December 2004, a total of 799 hypospadia cases were repaired at Meyer Children Hospital of Florence . Of these, 260 patients were unable to be reached by phone calls and were considered dropouts from the study. Finally, of the remaining 539 patients contacted, 300 refused to participate, mainly due to discomfort in discussing the topic over the phone. Two hundred thirty-nine patients consented to study participation and were interviewed. Follow-up via phone call occurred between 17 and 28 years after the first surgery.

**Figure 1.**
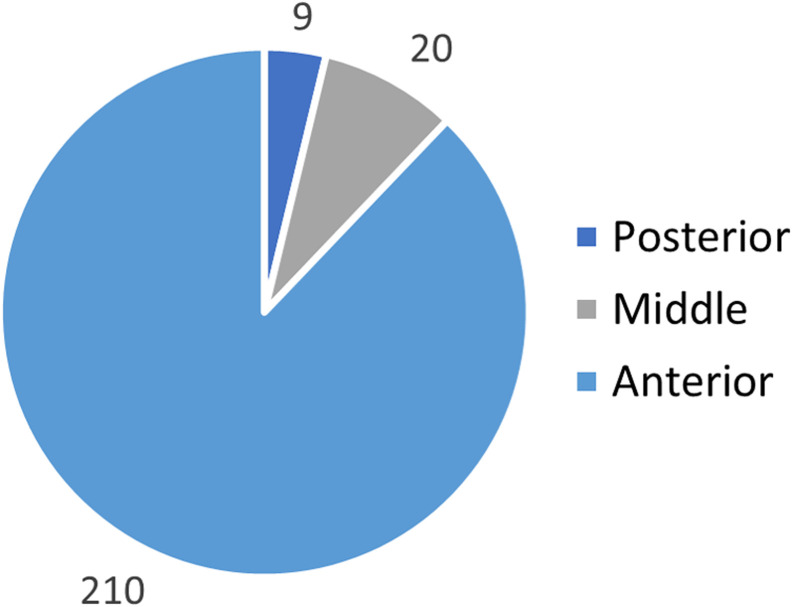
Hypospadias location.

**Figure 2.**
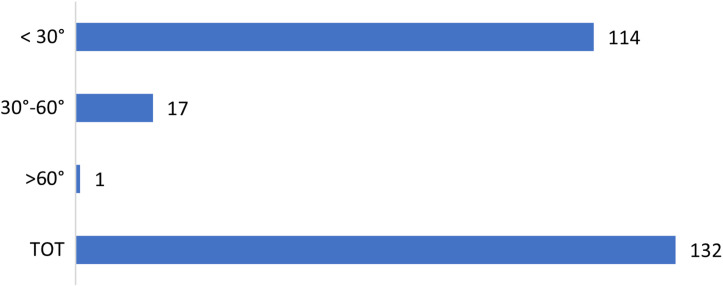
Penile curvature.

The 239 enrolled patients had an average age of 4.41 years old (SD ± 3.26; CI 95% 0.01) at the time of surgery. Most patients had an anterior localization of the hypospadias (210/239) (Figure 1), and in 132 patients, there was an association of various grades of penile curvature (Figure 2).

**Figure 3.**
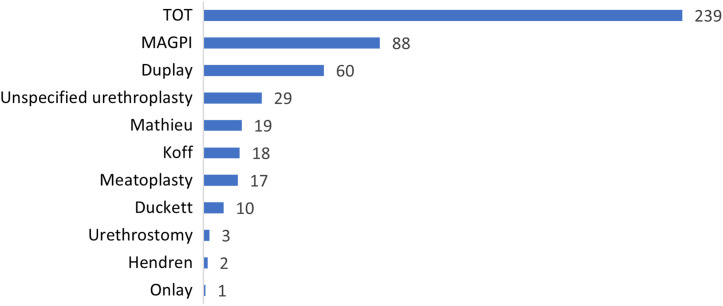
Surgical technique for hypospadias repair.

Data were collected regarding the type of surgery performed to treat hypospadias or penile curvature. The most frequent surgery for hypospadias repair was meatal advancement and glanduloplasty incorporated (MAGPI) (88/239), followed by Duplay (60/239) and Mathieu (19/239) (Figure 3).

**Figure 4.**
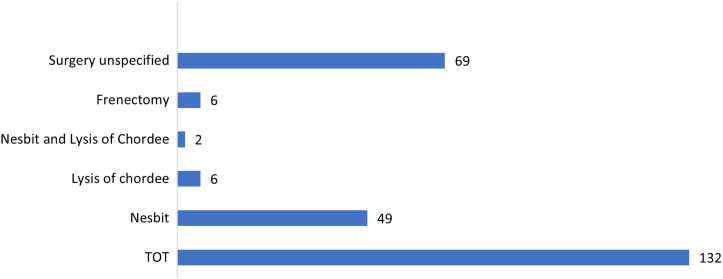
Surgical technique for penile curvature correction.

The most reported surgery for penile curvature repair was Nesbit’s technique (49/132), although surgical treatment was not specified in 69 patients (Figure 4).

**Figure 5.**
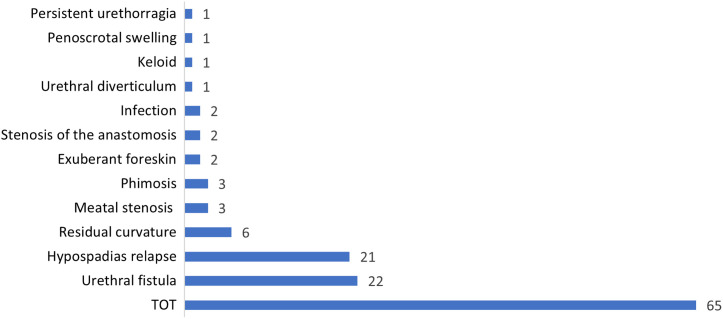
Short- and long-term complications.

The overall complication rate was 27% (n = 65), and the average time to the complications was 4.7 years (SD ± 5.6; 95% CI 0.02). The most common complications were hypospadias relapsing (n = 21) and urethral fistula (n = 22), followed by meatal or anastomosis stenosis, urethral diverticulum, residual curvature, infection, keloid, phimosis, exuberant foreskin, penoscrotal swelling, and persistent urethrorrhagia (Figure 5).

**Figure 6.**
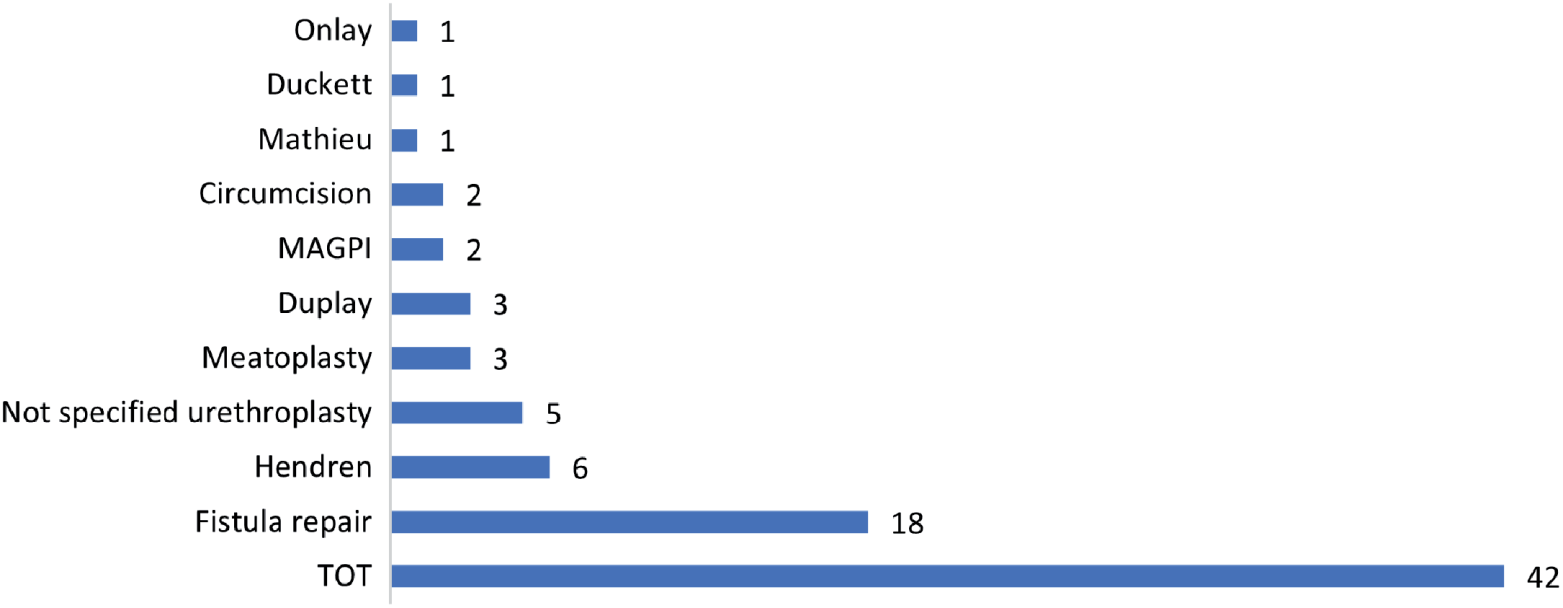
Surgical technique for hypospadias repair complications.

**Figure 7.**
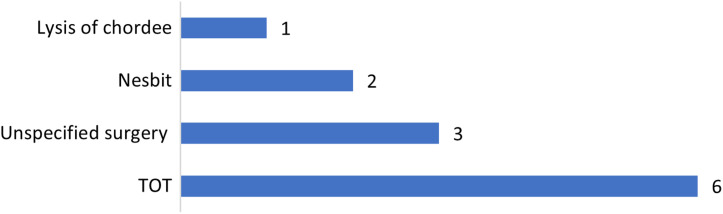
Surgical technique for residual penile curvature.

A total of 48 surgical procedures have been performed to treat complications. Of these, a suture of the fistula was performed in 18 patients, a redo urethoplasty in 19 cases (Figure 6), and penile curvature repair in 6 cases (Figure 7).

International Prostatic Symptoms Score, IIEF5 score, and HOSE score were calculated at follow-up. Patients who underwent reintervention reported a lower IPSS score than those who underwent only 1 surgery, 0.29 vs. 1.16 (Tble 1). No differences were reported between patients in IIEF5 score (Table 2) and HOSE-score (Table 3).

## Discussion

This study aims to evaluate long-term functional, sexual, and cosmetic outcomes in adult patients who underwent hypospadias repair during childhood between 1993 and 2004 at Meyer Children Hospital of Florence.

Hypospadias is one of the most common congenital genitourinary anomalies, and while modern hypospadiology has evolved considerably since the 1980s, significant challenges remain to be addressed.^4^

Both the malformation and its surgical outcome can affect adversely adolescent and adult life in their urinary, sexual, and cosmetic realm.^2,4^

There are few studies on long-term outcomes for adult patients treated in childhood, probably related to the lack of multidisciplinary transition and lifelong care teams and standardized assessment protocols.^2^

In our case series, an average age of 4.41 years old (±3.26 SD) was reported, although according to current European Pediatric Guidelines, the recommended age at surgery for primary hypospadias repair is usually 6-18 (24) months.^3^ The surgical result is at its best at this age, and the dangers of anesthetic and psychological aftereffects are low.^4^ If hypospadias correction is performed outside of this range, the patient may unintentionally be more at risk of complications.

Roy and Lerman discovered that infants younger than 3 months old had a higher incidence of laryngospasm.^6^ While it appears that surgeries done before the age of 5 are generally linked to a lack of perioperative memories,^1^ Lepore and Kesler found that 2- and 6-year-old boys having hypospadias repair experienced higher levels of hostility, aggression, and unfavorable interactions than kids having other kinds of surgeries.^7^ Hensle et al^8^ also observed a rise in surgical complications among people having hypospadias repaired.

Regarding the technique, even though more than 300 corrective procedures are described in the current literature, up to 10 of these have been performed in 12 years of practice at our center. Meatal advancement and glanduloplasty incorporated was the most used technique (88/239 patients), followed by Duplay (60/239), Mathieu (19/239) techniques, and the Koff procedure (18/239). The predilection for MAGPI, Duplay, and Mathieu techniques for mild hypospadias repair is also reported also by Chertin *et al*. in their case series of patients treated from 1978 to 1993.^9^ Conversely, since its introduction in 1994, tubularized incised plate urethroplasty (TIP) has become a popular technique for distal hypospadias worldwide, thanks to its easy execution, favorable outcomes, and low rate of complications.^10^

These days, urethral plates that are too narrow to be tubularized can be loosened by a median incision and then tubularized using the Snodgrass–Orkiszewski TIP technique. This is in accordance with European guidelines, which state that urethral plates that are wide can be tubularized using the Thiersch–Duplay technique.^3^ TIP is now the preferred method of treating both proximal and mid-range penile hypospadias, as well as distal hypospadias.^3^ Other frequently used procedures for distal hypospadias include urethral advancement and Mathieu.^3^ In primary and secondary repairs, it is advised to cover the exposed area of a deep plate incision with an inner preputial (or buccal) inlay graft.^11^ In cases of unhealthy or excessively narrow plates in patients with proximal hypospadias, the onlay technique using a preputial island flap is the recommended standard repair.^12^ One possibility for a one-stage repair is an onlay preputial graft.^13^ One- or 2-stage repairs are used if the urethral plate’s continuity cannot be maintained.^3^ A variant of the tubularized flap (Duckett tube), such as a tube-onlay, inlay–onlay flap, or onlay flap on albuginea, is typically used for single-stage to prevent urethral stricture; in other cases, the Koyanagi–Hayashi procedure is utilized.Due to improved results and a decreased incidence of ventral curvature recurrence, the 2-stage correction method has gained popularity in recent years.^14^

Regarding the surgical correction of penile curvature, Nesbit’s procedure appears to be the most widely adopted management in our case series (49/132), although for more than half of the patients, no accurate data were reported. Consequently, adequate conclusions cannot be drawn, but since most of the recorded curvatures were <30° (114/132), it seems reasonable to assume that most of the unspecified corrections may have been treated by lysis of chordee.

In fact, recommendations state that if penile curvature exists, it can be freed by degloving the penis (skin chordee) and by excising the chordee’s connective tissue on the ventral shaft, in as many as 70% of cases.^3^ Then, if there is still a curvature that has to be addressed, it is usually treated by orthoplasty, which is a variation of Nesbit’s procedure that may or may not involve elevating the neurovascular bundle.^3^ Ventral penile lengthening is advised in cases with severe curvature >45°, which is frequently accompanied by a short urethral plate that requires transection. This can be done with or without dorsal midline plication to prevent the penis from getting shorter.^3^

Concerning the complication rates, in our case studies, 27% of patients (65/239) reported short- or long-term ones, with an average time of 4.7 years since surgery and have required surgical treatment in 74% (50/65) of them. Consistent with the literature, urethral fistula (34%), hypospadias relapse (32%), and residual penile curvature (9%) were the most common complications.^3^

However, comparing our complication rate with other studies is challenging because this often correlates with the severity of hypospadias and the different surgical procedures performed,

which can lead to several outcomes. It is reasonable to assume that primary mild hypospadias repair is burdened by a lower rate of complications than severe ones or redo hypospadias repair. In this background, TIP appears to be a technique suitable for both distal and proximal hypospadias with a low complication rate, so its increasing use could potentially reduce complications and re-surgery rates in future studies.^3^ However, for redo hypospadias repairs, no definitive guidelines are available, and different procedures are tailored to the patient’s needs.^3^

As far as urinary function is concerned, it is a poorly analyzed aspect in studies on long-term outcomes after hypospadias repair. The IPSS^15^ was used in our series to evaluate, with a validated questionnaire, the potential development of inferior urinary symptoms (LUTSs).

Most patients did not complain of LUTSs, with an average IPSS overall score of 0.96. Unexpectedly, patients who underwent re-surgery reported a lower IPSS than those who underwent only one (0.29 vs. 1.16). It is conceivable that this finding is related to more severe cases or those that have developed major urinary complications such as fistulas, relapses, or urethral stenosis, which were then resolved by re-surgery, resulting in improved patient satisfaction. However, no firm conclusions may be drawn.

Although Perera et al^16^ described a lower urinary flow rate in adolescents who underwent hypospadias repair compared to age-matched controls (with no history of urological or neurological disorders), it did not seem to have a correlation with the development of LUTSs.

These similar findings may be due to the enrolled patients being young at the time of responding to the IPSS questionnaire, so the impact on urinary function in older patients may not be easily predicted based on this data; further studies with longer follow-ups are probably needed.

Unfortunately, in our study, objective data from uroflowmetries were not available for any enrolled patient, which indicates both a critical lack of adequate transition care from adolescence to adulthood and multidisciplinary lifelong care teams.

In terms of sexual function, previous studies have approached the assessment in various ways, leading to different findings. Chertin et al^9^evaluated sexual function using both unvalidated and validated questionnaires, including IIEF-5. They found a significant percentage of mild erectile dysfunction (ED) but nearly no impact on sexual desire and orgasmic function, with general satisfaction on sexual performance.^9^ Andersson et al,^17^ using an unvalidated questionnaire, reported a higher rate of episodic loss of erection, anejaculation, and uncertainty about ejaculatory function in adolescents who underwent hypospadias repair compared to age-matched controls. Jiao et al^18^ by unvalidated questionnaire have reported normal sexual function in a majority of patients; nevertheless, those with proximal hypospadias complained more erection and ejaculation problems than ones with distal hypospadias.

In our study, we have evaluated sexual function with the validated IIEF-5 questionnaire.^19^ Most patients did not report ED, with an average overall score of 24, and there was no difference noted for those who underwent re-surgery. The heterogeneous assessment of sexual function with validated and non-validated questionnaires makes comparisons between studies challenging.

Finally, penile cosmetic appearance can impact on psychosocial and psychosexual development.^20,21^ Psychosocial differences for proximal hypospadias have been described,^21^ as well as an increased risk of neurodevelopmental disorders in patients with hypospadias.^22^ However, few studies describe the outcomes from the point of view of adolescents.^23-26^

In our study, for the assessment of penile cosmetic appearance, we used HOSE, which focuses on meatal location and shape, urinary stream, curvature of erected penis, and presence of fistula.^27^ Most of the patients reported a normal cosmetic appearance of their penis with an average overall score of 15.67; only a few complained of a slightly abnormal conformation of the meatus or a mild residual curvature. No difference was observed for those who underwent re-surgery. These results are consistent with previous studies that used the HOSE score for cosmetic assessment. Jones et al^28^ reported that 80% of patients who underwent early surgery had an excellent esthetic outcome, and Aulagne et al^29^ had 21 out of 26 patients treated for severe hypospadias with a HOSE score equal to or greater than 14.

Although this represents a large Italian series conducted at a highly specialized pediatric center with long follow-up (occurred between 17 and 28 years after the first surgery), this study is not without limitations.

Many data were lost due to non-digitized records, and not all eligible patients answered the questionnaires. However, the response rate is acceptable (239 of 799 patients who underwent hypospadias repair), considering the study’s nature and the long-time interval between surgery and follow-up.

Moreover, the analyses were not stratified according to the degree of hypospadias severity and surgical techniques, and there was a low percentage of posterior hypospadias, typically associated with higher rates of complication and worse outcomes.

In conclusion, in this background, developing multidisciplinary teams in highly specialized centers would be crucial both to ensure adequate patient transition and long-life follow-up, and help reduce potential biases of adolescents in responding adequately to questionnaires, integrating objective assessment (uroflowmetry).

Our study shows that hypospadias repair in childhood can lead to excellent urinary, sexual, and cosmetic outcomes after the completion of psychosexual development. Further studies are needed to highlight potential differences between the surgical techniques performed, as well as the development of standardized assessment protocols by multidisciplinary transition and lifelong care teams.

## Figures and Tables

**Table 1.** IPSS at Follow-up

	IPSS Overall	IPSS Re-surgery	IPSS One Surgery
Average	0.96	0.29	1.16
SD	1.97	0.60	2.19
CI	0.01	0.01	0.01

IPSS, International Prostatic Symptoms Score; SD,standard deviation.

**Table 2.** IIEF5 Score at Follow-up

	IIEF5 Overall	IIEF5 Re-surgery	IIEF5 One Surgery
Average	24.10	24.09	24.11
SD	1.02	0.82	1.08
CI	0.00	0.20	0.01

IIEF5, International Index of Erectile Function-5; SD, standard deviation.

**Table 3.** HOSE Score Follow-up

	HOSE Overall	HOSE Re-surgery	HOSE One Surgery
Average	15.67	15.58	15.69
SD	0.45	0.51	0.43
CI	0.00	0.08	0.00

HOSE, hypospadias objective score; Evaluation SD, standard deviation.
